# Hospital-onset bacteremia and fungemia: An evaluation of predictors and feasibility of benchmarking comparing two risk-adjusted models among 267 hospitals

**DOI:** 10.1017/ice.2022.211

**Published:** 2022-10

**Authors:** Kalvin C. Yu, Gang Ye, Jonathan R. Edwards, Vikas Gupta, Andrea L. Benin, ChinEn Ai, Raymund Dantes

**Affiliations:** 1 Becton, Dickinson and Company, Franklin Lakes, New Jersey; 2 Centers for Disease Control and Prevention, Atlanta, Georgia; 3 Emory University School of Medicine, Atlanta, Georgia

## Abstract

**Objectives:**

To evaluate the prevalence of hospital-onset bacteremia and fungemia (HOB), identify hospital-level predictors, and to evaluate the feasibility of an HOB metric.

**Methods:**

We analyzed 9,202,650 admissions from 267 hospitals during 2015–2020. An HOB event was defined as the first positive blood-culture pathogen on day 3 of admission or later. We used the generalized linear model method via negative binomial regression to identify variables and risk markers for HOB. Standardized infection ratios (SIRs) were calculated based on 2 risk-adjusted models: a simple model using descriptive variables and a complex model using descriptive variables plus additional measures of blood-culture testing practices. Performance of each model was compared against the unadjusted rate of HOB.

**Results:**

Overall median rate of HOB per 100 admissions was 0.124 (interquartile range, 0.00–0.22). Facility-level predictors included bed size, sex, ICU admissions, community-onset (CO) blood culture testing intensity, and hospital-onset (HO) testing intensity, and prevalence (all *P* < .001). In the complex model, CO bacteremia prevalence, HO testing intensity, and HO testing prevalence were the predictors most associated with HOB. The complex model demonstrated better model performance; 55% of hospitals that ranked in the highest quartile based on their raw rate shifted to a lower quartile when the SIR from the complex model was applied.

**Conclusions:**

Hospital descriptors, aggregate patient characteristics, community bacteremia and/or fungemia burden, and clinical blood-culture testing practices influence rates of HOB. Benchmarking an HOB metric is feasible and should endeavor to include both facility and clinical variables.

Central-line–associated bloodstream infections (CLABSIs) have been a component of the US Centers for Medicare and Medicaid (CMS) national reporting programs since 2011. Despite setbacks in reduction of CLABSI related to the coronavirus disease 2019 (COVID-19) pandemic,^1^ CLABSI rates have decreased overall from historic prepandemic highs.^2^ Hospital-onset bacteremia and fungemia (HOB) has been suggested as a more comprehensive quality metric that may further enhance patient safety and infection prevention.^3–5^ An HOB metric could be algorithmically standardized using electronic health records (EHRs), which could mitigate manual attribution to a central line and potentially make HOB an objective quality measure that is optimized for reporting to incentive programs.^3–5^

Non-CLABSI bloodstream infections have also been shown to cause significant morbidity, mortality, and cost. Ridgway et al reported non-National Healthcare Safety Network (NHSN)–reportable bloodstream infection mortality of 23.6% and median cost of $86,927 (vs 6.7% and $62,929 for propensity-matched controls, respectively).^6^ Similar to CLABSI, an HOB metric would likely require risk adjustment for effective use in incentive programs. Advancements in accessibility of data in EHR systems may provide opportunities to standardize and improve risk adjustment by including factors beyond facility descriptors.^7–10^

In this study, we evaluated the prevalence of HOB events and identified hospital-level predictors associated with HOB using statistical models. Standardized infection ratios (SIRs), defined as the ratio of observed number of events (HOBs) divided by the predicted (expected) number of events, were calculated based on a simple model regression analysis (using easily extractable factors from EHRs as variables) and based on a complex model (which further included blood-culture practice variables). Hospital rankings based on the unadjusted HOB rate and on the model-based SIRs were compared.

## Methods

### Study design and population

This study was a retrospective, ecological study based on EHR microbiological, medication, and administrative data from adult patients (aged ≥18 years) admitted between October 1, 2015, and February 28, 2020, to any of 267 acute-care hospitals that contribute to the BD Insights and Research Database (Becton Dickinson, Franklin Lakes, NJ), which contains electronically captured laboratory, pharmacy, patient demographics, administrative data, and admission, discharge, and transfer data feeds.^11–13^ The distribution of hospitals in the database is similar to that of the United States as a whole.^13^ The study was approved as a limited retrospective data set for epidemiological analyses, exempted from consent by the New England Institutional Review Board and Human Subjects Research Committee (Wellesley, MA). It was conducted in compliance with Health Insurance Portability and Accountability Act requirements.

### Outcomes and definitions

For all analyses, the date of admission was considered day 1. An HOB event was defined when the following 2 requirements were met: (1) the first positive blood culture for a noncommensal organism (as defined by the Centers for Disease Control and Prevention^14^) within the hospital-onset (HO) period (day 3 or greater of hospitalization); and (2) receipt of a new antimicrobial (not previously administered in the prior 2 calendar days) with a first qualified antimicrobial day in the window period extending 2 calendar days before and 2 calendar days after the blood-culture collection (Supplementary Fig. S1 online). This definition is similar to the one used in the CDC Hospital Toolkit for Adult Sepsis Surveillance.^15^ At the time of this study, the definition of an NHSN HOB event had not been finalized.

Testing intensity was defined as the number of total blood cultures obtained in either the community-onset (CO) period, defined as the first 2 days of hospitalization, or the HO period, defined as day 3 or after hospital admission, divided by the number of total aggregate hospital admissions that had any blood culture performed. Conceptually, testing intensity reflects the cumulative blood cultures collected among admissions with any blood culture. Testing prevalence was defined as the number of admissions with any blood culture performed in the period (CO or HO) divided by the total number of aggregate admissions. Conceptually, this metric reflects the overall proportion of admissions with blood-culture testing.

Community-onset bacteremia or fungemia (COB) was defined as a first positive blood culture with a Centers for Disease Control and Prevention (CDC)-defined pathogen within the first 2 days of hospital admission. A subanalysis revealed that only 1.1% of COB admissions (N = 127,710) went on to have a subsequent nonmatching pathogen-positive blood culture during the HO period. The first positive blood culture positive for a noncommensal pathogen was used to designate an admission as COB or HOB.

### Statistical analysis

We approached the statistical analysis in 3 steps: (1) We identified the candidate variables that influence HOB rates using bivariate analysis. (2) We constructed simple and complex models for risk-adjusting HOB using SIRs derived from regression models and assess best model fit. (3) We compared hospital rankings using the raw, unadjusted HOB rates versus using risk adjustment from the models.

*Step 1.* HOB rates were calculated as the number of HOB events per 100 admissions for quarterly aggregated data. Bivariate analysis using general linear models was performed to explore the correlation between HOB rate and the following variables of interest:

1. *Clinical measures*. COB prevalence (rate of COB events per 100 admissions), percentage of intensive care unit (ICU) admissions (per all admissions), average length of hospital stay (LOS) among hospitalized patients (in days per admission), blood-culture prevalence, and intensity.
2. *Patient demographics:* Number of female patients per 100 admissions and percentage of patients in each age group (18–40, 41–64, 65–80, and >80 years).
3. *Facility characteristics:* bed size, medical school or non–medical school affiliation, and urban or rural status.

*Step 2.* To evaluate HOB rates with regression models, we used negative binomial regression methods to account for overdispersion of data, and we calculated incident rate ratios for relevant variables. We conducted 2 modeling analyses: a simple model and a complex model. The simple model used hospital-level variables for which data were easily obtained from EHRs and/or already reportable to the NHSN. Candidate variables considered in the simple model included facility- and hospital-level demographics of patients. Our complex model included variables in the simple model plus clinical practices of blood-culture testing divided into CO or HO blood-culture testing intensity and prevalence. To create the most parsimonious model, all continuous variables were partitioned into quartiles in the complex model.

For both models, we assessed higher model fit using Akaike information criteria (AIC) and Bayesian information criteria (BIC) based on the full data in the study cohort (3,498 quarters of aggregated data with 9,202,650 admissions). In addition, we used cross-validation methods in variable selection and confirmed that the full data and validation models had the same best set of variables in the final models.

*Step 3.* We compared hospital rankings based on the unadjusted (observed) HOB rate compared with rankings based on SIRs from the simple- and complex-adjusted models. We calculated the agreement test γ (gamma) statistic, Spearman correlation, and confidence intervals. We used the calculated 1-year SIR data (from 2019) as an example for comparison rankings. Finally, we compared rankings of the fourth quartile of unadjusted HOB rates of hospitals to their subsequent ranking using adjusted model SIRs. All analyses were conducted using SAS version 9.4 software (SAS Institute, Cary, NC).

## Results

The study included 9,202,650 patient admissions that were associated with 18,747 HOB events from 267 acute-care hospitals in the United States. Medical-school–affiliated hospitals accounted for 38.6% of hospitals, and urban facilities comprised 61.1% of all facilities. Among hospitals in this study, 33.7% had <100 beds, 42.3% had 100–300 beds, and 24.0.1% had >300 beds.

### HOB prevalence and bivariate analysis

Over the study period, the median rate of HOB events per 100 admissions was 0.124 (interquartile range [IQR], 0.00–0.22). HOB event rates did not significantly change over time (Table 1). Bivariate correlation analysis showed that all candidate variables in the analysis were correlated with HOB except for CO testing prevalence and percentage of patients aged 18–40 years (Table 1).

**Table 1.** Descriptive Statistics of HOB Rate and Bivariate Analysis Results

Variables	Admissions	HOB Events	HOB Rate per 100 Admissions					
			Lower Quartile	Median	Upper Quartile	Mean	SD	*P* Value
Overall	9,202,650	18,747	0.000	0.124	0.222	0.152	0.171	
**Year**								.1108
2015	345,954	844	0.052	0.140	0.250	0.175	0.168	
2016	1,719,915	3,741	0.000	0.128	0.234	0.160	0.174	
2017	2,049,727	3,953	0.027	0.126	0.213	0.147	0.152	
2018	2,314,695	4,498	0.000	0.121	0.218	0.147	0.163	
2019	2,265,115	4,585	0.000	0.121	0.221	0.151	0.190	
2020	507,244	1,126	0.000	0.124	0.230	0.156	0.161	
**COB rate per 100 admissions**								<.0001
1st quartile	2,106,482	3,026	0.000	0.049	0.144	0.098	0.148	
2nd quartile	2,726,188	5,512	0.067	0.140	0.229	0.162	0.140	
3rd quartile	2,516,053	6,190	0.070	0.153	0.254	0.185	0.185	
4th quartile	1,853,927	4,019	0.000	0.140	0.234	0.163	0.191	
**ICU admissions, %**								<.0001
Not reported	391,894	1,176	0.000	0.097	0.246	0.207	0.326	
1st quartile	1,604,752	2,268	0.000	0.063	0.154	0.099	0.154	
2nd quartile	2,639,107	4,113	0.071	0.137	0.208	0.149	0.112	
3rd quartile	2,283,445	4,838	0.047	0.137	0.240	0.159	0.144	
4th quartile	2,283,452	6,352	0.000	0.156	0.271	0.181	0.167	
**Test prevalence**								<.0001
1st quartile	2,539,201	4,005	0.000	0.084	0.185	0.118	0.130	
2nd quartile	2,570,229	4,899	0.053	0.138	0.219	0.152	0.133	
3rd quartile	2,533,628	6,268	0.074	0.160	0.255	0.188	0.180	
4th quartile	1,559,592	3,575	0.000	0.109	0.212	0.150	0.217	
**CO testing prevalence**								.1907
1st quartile	2,647,568	4,314	0.000	0.096	0.196	0.125	0.136	
2nd quartile	2,688,690	6,010	0.062	0.145	0.241	0.183	0.215	
3rd quartile	2,458,568	5,860	0.070	0.159	0.253	0.182	0.166	
4th quartile	1,407,824	2,563	0.000	0.087	0.182	0.118	0.143	
**HO testing prevalence**								<.0001
1st quartile	1,492,842	1,769	0.000	0.000	0.090	0.060	0.110	
2nd quartile	1,983,166	2,440	0.000	0.085	0.155	0.102	0.105	
3rd quartile	2,435,148	4,349	0.086	0.150	0.225	0.161	0.115	
4th quartile	3,291,494	10,189	0.157	0.245	0.354	0.284	0.227	
**Test intensity**								<.0001
Not calculated^a^	2,009	0	0.000	0.000	0.000	0.000	0.000	
1st quartile	1,344,262	2,329	0.000	0.000	0.131	0.083	0.166	
2nd quartile	1,831,097	2,602	0.000	0.093	0.172	0.111	0.113	
3rd quartile	2,588,338	4,551	0.080	0.148	0.227	0.160	0.112	
4th quartile	3,436,944	9,265	0.127	0.217	0.333	0.256	0.216	
**CO testing intensity**								<.0001
Not calculated^a^	2,009	0	0.000	0.000	0.000	0.000	0.000	
1st quartile	2,553,321	7,138	0.000	0.155	0.288	0.20	0.239	
2nd quartile	2,595,848	5,248	0.048	0.139	0.238	0.16	0.141	
3rd quartile	2,070,811	3,050	0.000	0.098	0.183	0.12	0.124	
4th quartile	1,980,661	3,311	0.000	0.110	0.203	0.13	0.144	
**HO testing intensity**								<.0001
Not calculated^a^	2,009	0	0.000	0.000	0.000	0.000	0.000	
1st quartile	754,629	448	0.000	0.000	0.068	0.05	0.095	
2nd quartile	1,762,768	2,074	0.000	0.097	0.159	0.11	0.112	
3rd quartile	2,821,882	4,717	0.087	0.151	0.223	0.16	0.105	
4th quartile	3,861,362	11,508	0.163	0.256	0.366	0.29	0.226	
**Average LOS**								<.0001
1st quartile	986,567	900	0.000	0.000	0.118	0.069	0.106	
2nd quartile	2,228,773	3,070	0.046	0.115	0.184	0.125	0.115	
3rd quartile	2,812,385	4,787	0.071	0.142	0.225	0.155	0.115	
4th quartile	3,174,925	9,990	0.105	0.230	0.354	0.259	0.245	
**Sex, female, %**								<.0001
1st quartile	2,558,016	7,124	0.078	0.173	0.305	0.220	0.237	
2nd quartile	2,750,286	5,687	0.044	0.134	0.238	0.156	0.140	
3rd quartile	2,344,363	3,770	0.000	0.117	0.192	0.133	0.131	
4th quartile	1,549,985	2,166	0.000	0.072	0.164	0.098	0.125	
**Patients aged 18–40 y, %**								.3053
1st quartile	1,441,315	2,895	0.000	0.083	0.213	0.145	0.238	
2nd quartile	2,527,501	4,340	0.056	0.131	0.209	0.143	0.119	
3rd quartile	3,043,248	6,505	0.068	0.146	0.246	0.168	0.137	
4th quartile	2,190,586	5,007	0.000	0.111	0.226	0.152	0.163	
**Patients aged 41–64 y, %**								<.0001
1st quartile	1,485,852	2,032	0.000	0.060	0.159	0.096	0.148	
2nd quartile	2,353,286	3,463	0.041	0.124	0.196	0.134	0.120	
3rd quartile	2,389,590	4,116	0.044	0.129	0.207	0.142	0.128	
4th quartile	2,973,922	9,136	0.067	0.204	0.347	0.236	0.231	
**Patients aged 65–80 y, %**								<.0001
1st quartile	2,303,940	5,775	0.000	0.131	0.261	0.170	0.170	
2nd quartile	2,619,330	5,123	0.040	0.134	0.225	0.148	0.132	
3rd quartile	2,641,306	4,779	0.041	0.128	0.214	0.142	0.126	
4th quartile	1,638,074	3,070	0.000	0.100	0.209	0.147	0.233	
**Patients aged >80 y, %**								<.0001
1st quartile	2,980,076	8,973	0.060	0.176	0.338	0.227	0.230	
2nd quartile	2,512,598	4,295	0.052	0.133	0.220	0.145	0.118	
3rd quartile	2,248,231	3,288	0.000	0.116	0.191	0.128	0.147	
4th quartile	1,461,745	2,191	0.000	0.073	0.178	0.108	0.141	
**Bed size (3-category)**								<.0001
< 100 beds	597,381	484	0.000	0.000	0.108	0.069	0.161	
100–300 beds	3,374,473	5,606	0.055	0.128	0.216	0.157	0.170	
> 300 beds	5,230,796	12,657	0.127	0.201	0.301	0.227	0.141	
**Bed size (refined grouping)**								<.0001
1–50 beds	147,420	78	0.000	0.000	0.000	0.054	0.200	
51–100 beds	449,961	406	0.000	0.000	0.144	0.085	0.109	
101–200 beds	1,395,467	1,889	0.000	0.106	0.186	0.129	0.129	
201–300 beds	1,979,006	3,717	0.085	0.151	0.242	0.193	0.205	
301–500 beds	3,051,740	5,877	0.113	0.168	0.252	0.196	0.127	
≥500 beds	2,179,056	6,780	0.201	0.285	0.379	0.300	0.147	
**Medical school affiliation**								<.0001
No	3,246,090	4,823	0.000	0.077	0.176	0.108	0.144	
Yes	5,956,560	13,924	0.097	0.168	0.282	0.212	0.185	
**Urban or rural**								<.0001
Rural	2,401,605	4,230	0.000	0.073	0.185	0.119	0.174	
Urban	6,801,045	14,517	0.059	0.143	0.242	0.170	0.166	

Note. CO, community-onset; COB, community-onset bacteremia; HO, hospital-onset; HOB, hospital-onset bacteremia; ICU, intensive care unit; LOS, length of stay; SD, standard deviation.

a Due to zero denominator.

### Variables associated with HOB: Simple model

Using the simple model, we identified the following as significant predictors: higher COB rate, longer average LOS, facilities with larger bed size, higher rate of ICU admissions, urban setting, higher percentage of patients aged 41–64 years, and higher percentage of male patients. The proportion of patients aged >80 years was negatively associated with HOB rates (Table 2 and Supplementary Table S1 online).

**Table 2.** HOB Predictors in the Simple Model^a^ With Estimated Incidence Rate Ratios^b^

Parameter	IRR (95% CI)^c^	*P* Value
COB rate per 100 admissions	1.39 (1.33–1.45)	<.0001
LOS, mean d	1.20 (1.18–1.22)	<.0001
**Bed size**		
1–100 beds		Reference
101–200 beds	1.40 (1.26–1.56)	<.0001
201–300 beds	1.85 (1.66–2.05)	<.0001
301–500 beds	1.98 (1.78–2.19)	<.0001
≥500 beds	2.23 (2.00–2.49)	<.0001
**ICU admissions, %**		
<2nd quartile		Reference
3rd quartile	1.12 (1.06–1.18)	<.0001
4th quartile	1.19 (1.12–1.26)	<.0001
Not reported	1.64 (1.49–1.79)	<.0001
**Sex, female, %**	0.99 (0.99–1.00)	0.0017
**Patients aged 41-64 y, %**	1.02 (1.01–1.02)	<.0001
**Patients aged >80 y, %**	0.97 (0.96–0.97)	<.0001
**Urban or rural status**		
Rural		Reference
Urban	1.08 (1.03–1.14)	0.0013

Note. CI, confidence interval; COB, community-onset bacteremia; HOB, hospital-onset bacteremia; ICU, intensive care unit; IRR, incidence rate ratio; LOS, length of stay.

a Goodness-of-fit statistics: Akaike information criteria, 13,409; Bayesian information criteria, 13,501.

b For model replication purposes, regression coefficients and standard errors are presented in Supplementary Table S1 (online).

c Estimated increase in HOB relative to the reference. As an example, for hospitals with 101–200 beds, the IRR was 1.40. Holding other variables constant in the model, hospitals with 101–200 beds are expected to have a HOB rate 1.40 times greater (40% greater) than the hospitals with 1–100 beds.

### Variables associated with HOB: Complex model

Predictors associated with higher HOB rates in the complex model were increased COB, increased HO blood-culture testing intensity, increased HO test prevalence, fourth-quartile % ICU admission, fourth-quartile LOS, larger bed size, and percentage of patients aged 41–64 years (all *P* < .001) (Table 3 and Supplementary Table S2 online). The most influential factor was HO testing intensity. Compared with hospitals in the first quartile of HO test intensity, HOB rates per 100 admissions were expected to be higher by a factor of 1.58 for hospitals in the second quartile (ie, 58% higher), by a factor of 1.93 for hospitals in the third quartile (ie, 93% higher), and by a factor of 2.39 for hospitals in the fourth quartile (ie, 139% higher). Similar interpretations applied to other model predictors. Variables negatively associated with HOB rates were increased CO blood-culture testing intensity and percentage of patients >80 years. The variable of urban (vs rural) did not remain significant and was therefore not included in the final complex model. Medical-school affiliation was not significant in either model.

**Table 3.** HOB Predictors in the Complex Model^a^ with Estimated Incidence Rate Ratios^b^

Parameter	IRR (95% CI)^c^	*P* Value
**COB rate per 100 admissions**		
1st quartile		Reference
2nd quartile	1.25 (1.17–1.33)	<.0001
3rd quartile	1.44 (1.35–1.54)	<.0001
4th quartile	1.52 (1.41–1.63)	<.0001
**HO test intensity**		
1st quartile		Reference
2nd quartile	1.58 (1.41–1.76)	<.0001
3rd quartile	1.93 (1.72–2.16)	<.0001
4th quartile	2.39 (2.12–2.69)	<.0001
**CO test intensity**		
1st quartile		Reference
2nd quartile	0.83 (0.79–0.87)	<.0001
3rd quartile	0.76 (0.72–0.81)	<.0001
4th quartile	0.74 (0.70–0.79)	<.0001
**HO test prevalence**		
<2nd quartile		Reference
3rd quartile	1.25 (1.18–1.33)	<.0001
4th quartile	1.39 (1.30–1.48)	<.0001
**ICU admissions, %**		
≤3rd quartile		Reference
4th quartile	1.11 (1.05–1.16)	<.0001
Not reported	1.61 (1.48–1.74)	<.0001
**Mean LOS**		
≤3rd quartile		Reference
4th quartile	1.15 (1.09–1.21)	<.0001
**Bed size**		
01–100 beds		Reference
101–200 beds	1.25 (1.12–1.39)	<.0001
201–300 beds	1.49 (1.34–1.65)	<.0001
301–500 beds	1.43 (1.29–1.59)	<.0001
≥500 beds	1.42 (1.27–1.59)	<.0001
**Patients aged 41–64 y, %**		
1st quartile		Reference
2nd quartile	1.12 (1.04–1.20)	0.0015
3rd quartile	1.15 (1.07–1.24)	<.0001
4th quartile	1.38 (1.28–1.49)	<.0001
**Patients aged >80 y, %**		
1st quartile		Reference
2nd quartile	0.84 (0.79–0.89)	<.0001
3rd quartile	0.79 (0.74–0.84)	<.0001
4th quartile	0.83 (0.77–0.90)	<.0001

Note. AIC, Akaike information criteria; BIC, Bayesian information criteria; CI, confidence interval; CO, community onset; COB, community-onset bacteremia; HO, hospital onset; HOB, hospital-onset bacteremia; ICU, intensive care unit; IRR, incidence rate ratio; LOS, length of stay.

a Goodness-of-fit statistics: AIC = 12,882, BIC = 13,042. For the complex model we calculated a 4% reduction in AIC and a 3.4% reduction in BIC compared with the simple model, which indicates an improvement in model fit.

b For model replication purposes, regression coefficients and standard errors are presented in Supplementary Table S2 (online).

c Estimated increase in HOB relative to the reference. For example, compared to those hospitals in the first quartile of HO test intensity, hospitals in the second quartile were 1.58 times higher (58% higher) in HOB rate per 100 admissions.

In terms of model fit, the complex model afforded a 4% reduction in AIC and a 3.4% reduction in BIC compared with the simple model, indicating improvement in model fit.

### Comparison of hospital rankings

**Fig. 1.**
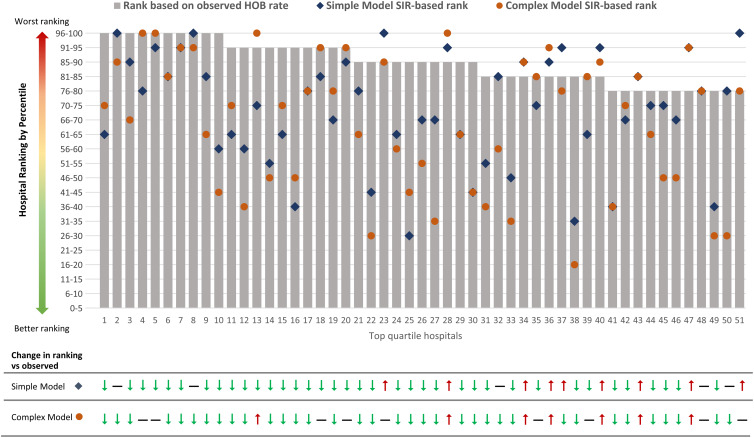
Hospital rankings for top-quartile hospitals (designated 1–51) based on observed HOB rates compared with the simple- and complex-model–derived SIR ranking.^a^ Gray bars represent rank of the top quartile of hospitals based on observed unadjusted HOB rate per 100 admissions. Blue diamonds represent the simple model SIR-based rank. Orange circles represent the complex model SIR-based rank. ^a^For example, hospital 10 (of 51) is in the top 95th percentile based on observed (unadjusted) HOB; it drops in rank with simple model SIR adjustment to the 56th–60th percentile and further decreases to the 41st–45th percentile in the complex model SIR-adjusted model. Note that among the 51 hospitals, some also increased in rank after the complex-model SIR adjustment (ie, hospitals 13, 28, 34, 36, 40, 43, 47). Full movements of rankings in all 4 quartiles are summarized in Supplementary Table S3 (online).

We compared rankings of hospitals with the highest (“worst performing” or fourth quartile) observed HOB rates (hospitals 1–51). Risk adjustment after applying the simple- and complex-model–derived SIRs resulted in changes in hospital rankings to unique and different degrees compared to observed HOB rankings (Fig. 1 and Supplementary Table S3 online).

Agreement test of the rankings showed that the unadjusted HOB rate had a strong ordinal association with rankings of the simple model SIR: γ statistic, 0.72 (95% CI, 0.64–0.81) and Spearman correlation, 0.67 (95% CI,0. 60–0.75). The strength of association between the unadjusted HOB event rate and complex-model SIR was noticeably lower (γ statistic, 0.56; 95% CI, 0.45–0.67) and Spearman correlation, 0.52 (95% CI, 0.42–0.63). These findings demonstrate the differential adjustment afforded by the complex model (Supplementary Table S4 online).

We demonstrated potential real-world application of risk adjustment by quantifying changes in the top 51 hospitals (fourth quartile) of observed HOB rate compared with complex-model SIR rank. Only 23 hospitals (45%) remained in the fourth quartile. The other 28 hospitals (55%) moved to lower-rank quartiles following risk adjustment in the complex model (Supplementary Fig. S2 online and Supplementary Table S3 online).

## Discussion

SIRs are used by the CDC, CMS, and other stakeholders to express performance for measures of inpatient care, including healthcare-associated infections.^2,16,17^ For the simple-model SIR we included hospital-level variables already reported to the NHSN or available in EHRs. The complex model contained the same variables as the simple model; however, all continuous variables were changed to categorical quartiles to maximize the efficiency of continuous covariate support. The changes efficiently covered the range of values without having undue influence from extreme values and created the most parsimonious model. The complex model also included categorical designations of clinical blood-culture testing practices, represented by HO and CO blood-culture testing intensity and prevalence. By doing so, we included signals of clinical acumen for deciding when to obtain blood cultures. For example, volume of blood-culture burden and time to documentation of pathogen clearance are standard of practice for endocarditis and other invasive infections.

Administrative and financial claims data (ie, *International Classification of Disease* and/or diagnosis-related group codes) for risk adjustment are potentially burdensome for hospitals to send and are not finalized until discharge and billing are completed. In their absence, blood-culture testing intensity and prevalence may serve as a more readily accessible signal of different patient case mixes at risk for HOB. Of the 51 hospitals representing the top 25th percentile of observed HOB rates, 28 (55%) shifted to a lower rank when these factors were accounted for, including 1 hospital that shifted from the highest observed HOB rate to the lowest quartile. Others have used clinical variables to risk-adjust assessment of infection events^10^ while respecting the need for standardization.^7^ The challenge is creating a standardized metric for HOB that is feasible while balancing the need for using clinical factors for appropriate risk adjustment. To that end, we created the simple model to help inform the complex model that included all variables from the former plus clinical blood-culture testing practices. The simple model was ultimately nested within the complex model.

Epidemiologically, unadjusted hospital infection rates will not account for different patient case mixes among facilities,^8–10^ suggesting that more nuanced risk adjustment should be explored. To this end, we endeavored to show preferential adjustment by the complex-model SIR through a 3-step process: (1) We identified the predictors of HOB. (2) We assessed the best model fit among simple-model versus complex-model SIRs. (3) We demonstrated differentiated SIR ranking between the 2 models and observed HOB rates. For due diligence, we quantified ranking changes applied to the top observed HOB quartile of hospitals and showed higher correlation with simple-model SIR than complex-model SIR, which suggests that the complex-model SIR is “adjusting more” while including clinical testing variables. The model-fit statistics showed that the complex model afforded a noticeable reduction in AIC and BIC compared with the simple model. This comparison provided statistical support for the use of the predictors incorporated in the complex model. HO intensity and prevalence were strongly associated with HOB. Blood-culture intensity and prevalence might serve as proxy signals for different clinical case mixes; they are more readily extractable electronic data elements compared to a breakdown of infected patients. For example, neutropenia- or malignancy-related bacteremia, discitis, and prosthetics-associated bacteremia are conditions that often require documentation and clearance of bacteremia,^18–20^ but the specific patient case mix may be difficult to capture electronically. Although we did include proxies for higher-level care such as medical school affiliation (which fell out of significance in both models), bed volume, and ICU admissions, the blood-culture HO intensity and prevalence practices demonstrated higher correlation with HOB and are perhaps a better representation of suspected patient types that warrant more frequent blood cultures and, therefore, may affect baseline attribution of HOB.^10^ Notably, the complex model also includes COB incidence, which was highly correlated in the multivariate analysis with HOB. Aside from perhaps representing another signal of more at-risk patient demographics, higher community prevalence of bloodstream pathogens may also be more directly associated with HOB, as has been found in other studies of correlations between community prevalence and subsequent higher risk of HO infection of that pathogen, such as *Clostridiodes difficile* infection (CDI).^21^

Widespread use of an HOB measure raises the potential of influencing blood-culture practices. Blood-culture testing in the “CO” timeframe may increase to avoid an HOB designation. In our multivariate analysis, a higher CO (first 2 days of admission) testing intensity was associated with lower HOB (ie, negative or protective association). This potential is not necessarily problematic because faster identification of bacteremia may facilitate definitive therapy. The danger is that increased CO testing more broadly and in patients not strongly suspected of having a COB infection—that is, increasing CO testing prevalence—could also occur. However, CO testing prevalence was not a significant factor associated with HOB (either negative or positive) in our model, and that finding may ease concerns of testing-practice changes meant to manipulate a reportable metric. In short, the complex model may mitigate potential shifts in blood-culture practices by incorporating and accounting for those changes in the model itself.

Quality measures have the potential to impact the use of diagnostic microbiology tests. In one study, reductions in NHSN-reportable CDI events occurred after changes in their testing algorithm.^22^ If an HOB metric influences changes in testing, incorporating COB prevalence in the same model that includes blood-culture acquisition practices may help adjust the HOB measure to reflect testing changes. Furthermore, delineating baselines of HOB, COB, and blood-culture testing intensity and prevalence will enable future analyses that endeavor to improve bacteremia care and blood-specimen collection practices. Additionally, the clinical and financial consequences of growth of commensals and/or improper blood-culture technique (ie, blood-culture ‘contaminants’) are not insignificant.^23^ Therefore, the value of the complex model may be not only in benchmarking HOB but also in promoting improvements in diagnostic blood culture practices, technique, and resource stewardship.

This study had several limitations. First, several permutations of HOB definitions have been explored recently; thus, the HOB definition used here may not precisely match the NHSN definition. The clinical preventability of an HOB event was not within the scope of this project and is being explored by other complementary projects. Prior studies have looked at HOB but have allowed contaminants and/or for COB and HOB in the same admission.^3^ Because prospective surveillance for HOB does not currently exist, any initial HOB definition is likely to undergo refinement as additional scientific knowledge is gained. In addition, some HOB events were “missing” because some hospitals do not map all units to NHSN categories; therefore, some ICU admissions were not reported (labeled as “not reported” in Tables 2 and 3). Lastly, although we do not present a “gold standard” metric, we do present optics on improvement of existing SIR risk adjustment using available and current real-world data. As data interoperability capabilities improve, infection prevention-related reporting should be streamlined to minimize manual reporting burden and prioritize activities such as education and root-cause analyses. The balance of transforming electronically capturable data into meaningful metrics will help facilitate fair assessment and improve patient care.

The risk adjustment achieved with the complex model is distinct and uniquely distinguishes differential HOB ranking when compared with unadjusted rates. In addition to incorporating factors included in the simple model, the complex model includes differences in blood-culture testing practices that, in aggregate, may improve model fit, may achieve lower estimation error, and may more accurately reflect fluctuating patient case mixes at risk for HOB than some broad facility-level categories. More specifically, facility descriptors, patient characteristics, COB prevalence, and different aspects of blood-culture testing intensity and prevalence during the HO and CO periods were significant factors associated with HOB incidence. A national HOB metric should endeavor to include these characteristics.

## Supplementary material

For supplementary material accompanying this paper visit https://doi.org/10.1017/ice.2022.211. click here to view supplementary material
