# Supplementary material for: Hospital-onset bacteremia and fungemia: An evaluation of predictors and feasibility of benchmarking comparing two risk-adjusted models among 267 hospitals
Source: Infect Control Hosp Epidemiol. 2022 Sep 9;43(10):1317–25. doi: 10.1017/ice.2022.211 (PMC9588439; doi:10.1017/ice.2022.211)
Supplement: Supplementary file 1 [file S0899823X22002112sup001.docx]

**Supplementary Materials**

**Hospital-Onset Bacteremia and Fungemia: An Evaluation of Predictors and Feasibility of Benchmarking Comparing Two Risk-Adjusted Models Among 267 Hospitals**

Kalvin C. Yu MD^1^, Gang Ye PhD^1^, Jonathan R. Edwards MStat^2^, Vikas Gupta PharmD^1^, Andrea L. Benin MD^2^, ChinEn Ai PhD^1^, Raymund Dantes MD, MPH^2,3^

^1^Becton, Dickinson and Company, 1 Becton Drive, Franklin Lakes, New Jersey, USA

^2^Centers for Disease Control and Prevention, Atlanta, Georgia, USA

^3^Emory University School of Medicine, Atlanta, Georgia, USA

**Supplementary Figure S1.** New antibiotics starting day in relation to HOB culture day (day 0). Qualified antibiotic days (QAD) were defined as an antimicrobial started within 2 calendar days before or after the day of collection for a blood culture positive HOB event. A QAD of “7” indicates there were 7 days of antimicrobial therapy for either a CDC-defined bacterial or *Candida* spp. pathogen that was started within 2 days before or after the collection date of the positive blood culture. Data is shown for a subset of 85 hospitals.


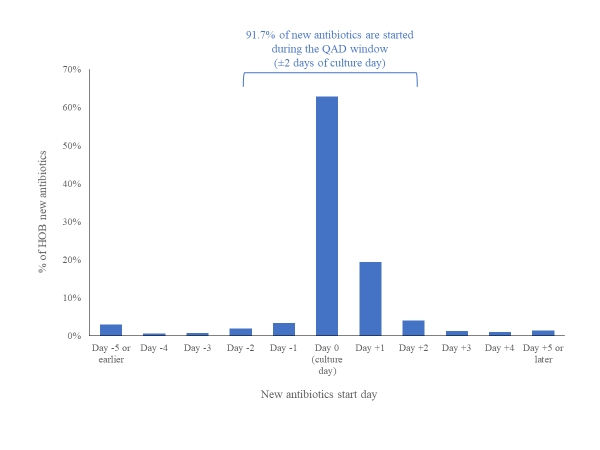


**Supplementary Figure S2.** Ranking change of hospitals with the highest unadjusted HOB event rates (4th quartile in ranked HOB rate) based on Complex Model-adjusted SIR. A more granular breakdown of these hospitals and the rank adjustment after applying Simple and Complex SIR is included in Supplementary Table S3.


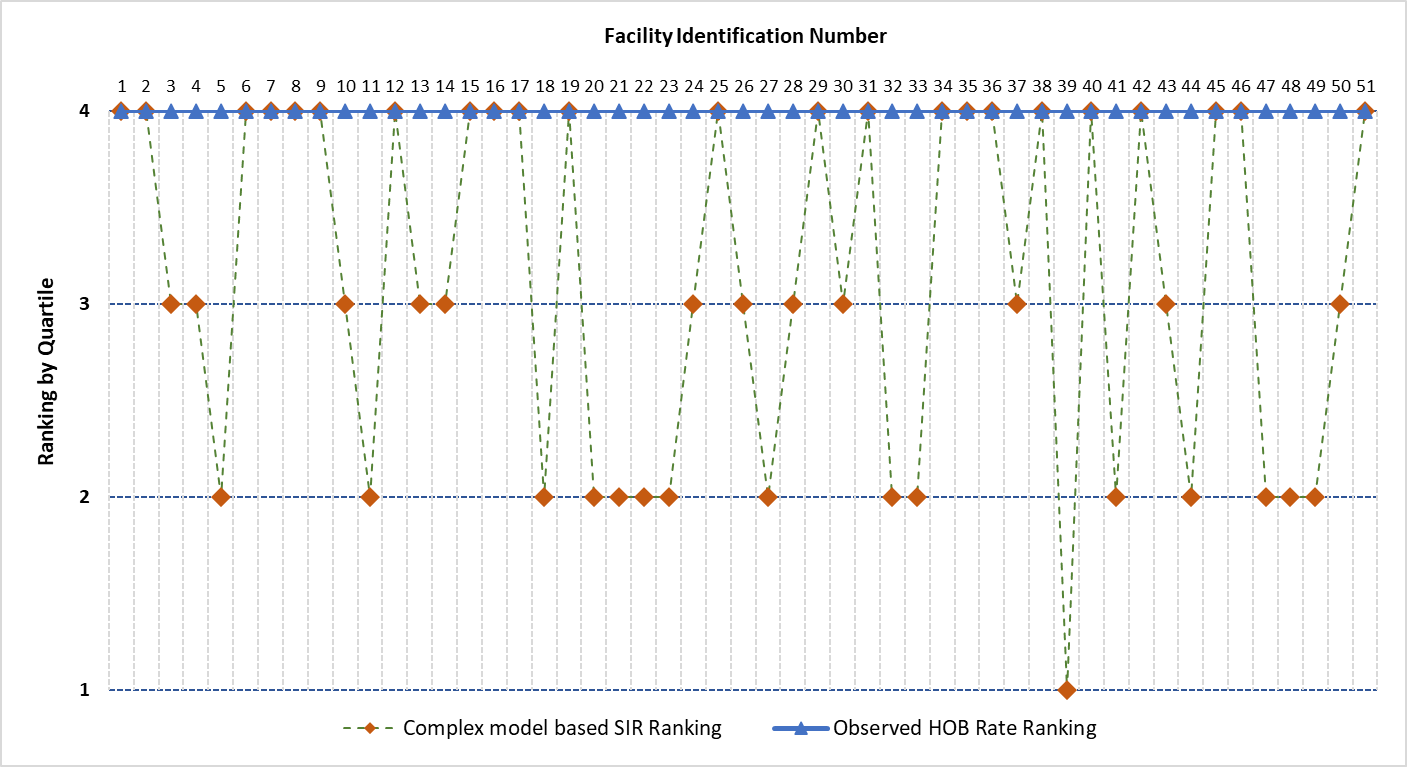


**Supplementary Table S1.** Regression Coefficients and Standard Errors for the Simple Model.

| **Parameter** | **Regression Coefficient**  **(in Logarithm Scale)** | **Standard Error** |
| --- | --- | --- |
| **Intercept** | -3.318 | 0.302 |
| **COB rate per 100 admissions** | 0.328 | 0.021 |
| **Mean LOS** | 0.184 | 0.010 |
| **Bed size** |  |  |
| 1-100 |  |  |
| 101-200 | 0.337 | 0.055 |
| 201-300 | 0.613 | 0.054 |
| 301-500 | 0.681 | 0.052 |
| 500+ | 0.803 | 0.056 |
| **% ICU admissions** |  |  |
| < 2nd quartile |  |  |
| 3rd quartile | 0.112 | 0.028 |
| 4th quartile | 0.171 | 0.030 |
| Not reported | 0.493 | 0.047 |
| **% female** | -0.009 | 0.003 |
| **% of patients aged 41-64 years** | 0.016 | 0.003 |
| **% of patients aged >80 years** | -0.032 | 0.003 |
| **Urban/rural status** |  |  |
| Rural |  |  |
| Urban | 0.081 | 0.025 |

COB, community-onset bacteremia; HOB, hospital-onset bacteremia; ICU, intensive care unit; LOS, length of stay

**Supplementary Table S2.** Regression Coefficients and Standard Errors for the Complex Model.

| **Parameter** | **Regression Coefficient**  **(in Logarithm Scale)** | **Standard Error** |
| --- | --- | --- |
| **Intercept** | -3.115 | 0.073 |
| **COB rate per 100 admissions** |  |  |
| 1^st^ quartile |  |  |
| 2^nd^ quartile | 0.221 | 0.032 |
| 3^rd^ quartile | 0.366 | 0.033 |
| 4^th^ quartile | 0.416 | 0.038 |
| **HO test intensity** |  |  |
| 1^st^ quartile |  |  |
| 2^nd^ quartile | 0.455 | 0.057 |
| 3^rd^ quartile | 0.658 | 0.059 |
| 4^th^ quartile | 0.869 | 0.061 |
| **CO test intensity** |  |  |
| 1^st^ quartile |  |  |
| 2^nd^ quartile | -0.188 | 0.027 |
| 3^rd^ quartile | -0.271 | 0.032 |
| 4^th^ quartile | -0.295 | 0.032 |
| **HO test prevalence** |  |  |
| < 2^nd^ quartile |  |  |
| 3^rd^ quartile | 0.222 | 0.031 |
| 4^th^ quartile | 0.327 | 0.034 |
| **%ICU admissions** |  |  |
| ≤ 3^rd^ quartile |  |  |
| 4^th^ quartile | 0.100 | 0.025 |
| Not reported | 0.474 | 0.042 |
| **Mean LOS** |  |  |
| ≤ 3^rd^ quartile |  |  |
| 4^th^ quartile | 0.140 | 0.026 |
| **Bed size** |  |  |
| 01-100 |  |  |
| 101-200 | 0.225 | 0.055 |
| 201-300 | 0.396 | 0.055 |
| 301-500 | 0.358 | 0.054 |
| 500+ | 0.351 | 0.057 |
| **% of patients aged 41-64 years** |  |  |
| 1^st^ quartile |  |  |
| 2^nd^ quartile | 0.111 | 0.035 |
| 3^rd^ quartile | 0.141 | 0.036 |
| 4^th^ quartile | 0.325 | 0.038 |
| **% of patients aged >80 years** |  |  |
| 1^st^ quartile |  |  |
| 2^nd^ quartile | -0.175 | 0.029 |
| 3^rd^ quartile | -0.234 | 0.033 |
| 4^th^ quartile | -0.183 | 0.038 |

CO, community-onset; COB, community-onset bacteremia; HO, hospital-onset; HOB, hospital-onset bacteremia; ICU, intensive care unit; LOS, length of stay.

**Supplementary Table S3.** Summary of Rank Changes of all 4 Quartiles of Hospitals in the Simple and Complex Models. Gray shading indicates rankings that were unchanged between the observed rank and the models. Data are shown as % (number of hospitals).

| **Observed HOB Rank (N)** | **Simple Model, % (N)** | | | | **Complex Model, % (N)** | | | |
| --- | --- | --- | --- | --- | --- | --- | --- | --- |
|  | Rank 1 | Rank 2 | Rank 3 | Rank 4 | Rank 1 | Rank 2 | Rank 3 | Rank 4 |
| Rank 1: Quartile 1 (50) | 72% (36) | 24% (12) | 04% (2) | - | 66% (33) | 24% (12) | 08% (4) | 02% (1) |
| Rank 2: Quartile 2 (51) | 24% (12) | 35% (18) | 25% (13) | 16% (8) | 25% (13) | 20% (10) | 27% (14) | 27% (14) |
| Rank 3: Quartile 3 (51) | 02% (1) | 25% (13) | 35% (18) | 37% (19) | 06% (3) | 27% (14) | 41% (21) | 25% (13) |
| Rank 4: Quartile 4 (51) | - | 16% (8) | 37% (19) | 47% (24) | 02% (1) | 29% (15) | 24% (12) | 45% (23) |

**Supplementary Table S4.** Measures of Ranked Association Between the Models and Unadjusted HOB Rate.

| **Model comparison** | **Agreement test for rankings** | |
| --- | --- | --- |
|  | **Gamma statistic**  **(95% CI)** | **Spearman correlation (95% CI)** |
| Unadjusted HOB rate vs Simple Model SIR | 0.72 (0.64-0.81) | 0.67 (0.60-0.75) |
| Unadjusted HOB rate vs Complex Model SIR | 0.56 (0.45-0.67) | 0.52 (0.42-0.63) |

Note. CI, confidence interval; HOB, hospital-onset bacteremia; SIR, standardized infection ratio.
